# Erythrocytes of the common carp are immune sentinels that sense pathogen molecular patterns, engulf particles and secrete pro-inflammatory cytokines against bacterial infection

**DOI:** 10.3389/fimmu.2024.1407237

**Published:** 2024-06-14

**Authors:** Jovana Majstorović, Jiří Kyslík, Katarzyna Klak, Magdalena Maciuszek, Justin T. H. Chan, Tomáš Korytář, Astrid S. Holzer

**Affiliations:** ^1^ Laboratory of Fish Protistology, Institute of Parasitology, Biology Centre, Czech Academy of Sciences, České Budějovice, Czechia; ^2^ Faculty of Science, University of South Bohemia, České Budějovice, Czechia; ^3^ Department of Evolutionary Immunology, Institute of Zoology and Biomedical Research, Faculty of Biology, Jagiellonian University, Krakow, Poland; ^4^ Doctoral School of Exact and Natural Sciences, Jagiellonian University, Krakow, Poland; ^5^ Fish Health Division, Veterinary University of Vienna, Vienna, Austria

**Keywords:** red blood cell (RBC), teleost fish, cytokines, bacteria, *Aeromonas hydrophila* (*A. hydrophila*), *Cyprinus carpio*, inflammation, engulfment

## Abstract

**Introduction:**

Red blood cells (RBCs), also known as erythrocytes, are underestimated in their role in the immune system. In mammals, erythrocytes undergo maturation that involves the loss of nuclei, resulting in limited transcription and protein synthesis capabilities. However, the nucleated nature of non-mammalian RBCs is challenging this conventional understanding of RBCs. Notably, in bony fishes, research indicates that RBCs are not only susceptible to pathogen attacks but express immune receptors and effector molecules. However, given the abundance of RBCs and their interaction with every physiological system, we postulate that they act in surveillance as sentinels, rapid responders, and messengers.

**Methods:**

We performed a series of *in vitro* experiments with *Cyprinus carpio* RBCs exposed to *Aeromonas hydrophila*, as well as *in vivo* laboratory infections using different concentrations of bacteria.

**Results:**

qPCR revealed that RBCs express genes of several inflammatory cytokines. Using cyprinid-specific antibodies, we confirmed that RBCs secreted tumor necrosis factor alpha (TNFα) and interferon gamma (IFNγ). In contrast to these indirect immune mechanisms, we observed that RBCs produce reactive oxygen species and, through transmission electron and confocal microscopy, that RBCs can engulf particles. Finally, RBCs expressed and upregulated several putative toll-like receptors, including *tlr4* and *tlr9*, in response to *A. hydrophila* infection *in vivo*.

**Discussion:**

Overall, the RBC repertoire of pattern recognition receptors, their secretion of effector molecules, and their swift response make them immune sentinels capable of rapidly detecting and signaling the presence of foreign pathogens. By studying the interaction between a bacterium and erythrocytes, we provide novel insights into how the latter may contribute to overall innate and adaptive immune responses of teleost fishes.

## 1 Introduction

Blood is composed of both a liquid and an insoluble cellular fraction: plasma and cells of hematopoietic origin, respectively. Blood circulates and interacts with every physiological system: e.g., to supply nutrients absorbed in the digestive system, for hormonal regulation via the endocrine system, to eliminate waste via the kidneys, and notably for immune defense. Conventionally, leukocytes (white blood cells, WBCs) are the mediators of immunity whereas erythrocytes (red blood cells, RBCs) maintain homeostasis via a non-immunological role by distributing oxygen from the lungs to tissues, returning to the lungs to eliminate the CO_2_ produced in respiration, and by regulating blood pH. Their immunological activity extends to transporting immune complexes of antibodies bound to both self-antigens and foreign antigens destined for homeostatic clearance in the spleen and liver (1, 2). However, in the past decade, our understanding of the immune competence of these cells is changing with growing interest and new discoveries in immunology of nucleated non-mammalian RBCs.

Recent studies on both nucleated (3) and anucleate (4–8) RBCs indicate that they are not passive bystanders but rather participate in host defense and communicate with white blood cells to combat pathogens (9). Focusing on nucleated RBCs, beyond binding immune complexes via a complement receptor, both chicken and fish erythrocytes can detect lipopolysaccharide (LPS) and the TLR3 agonist polyinosinic:polycytidylic acid (poly(I:C)) which mimics viral dsRNA (10, 11). Detection of microbe-associated molecular patterns (MAMPs) may be via pattern recognition receptors (PRRs) such as toll-like receptors (TLRs) expressed by the RBCs (11). The subsequent expression of chemokine (C-C motif) ligand 4 (*ccl4*) (11), interleukin-8 (*il8*) (12), or type 1 interferons and other inflammatory cytokines (13), suggest that nucleated RBCs participate in immune responses. Their responses may not be limited to communication with other immune cells because RBCs also produce antimicrobial peptides (13), and potentially engulf and inactivate microbes by producing reactive oxygen species (ROS) (14, 15).

In contrast to WBCs, RBCs are rich in iron. In addition, the nucleated nature of ectotherm RBCs also makes them susceptible to a variety of pathogens (16). In fish, they are directly targeted by viral (17), bacterial (18) and parasitic (19) pathogens. Fish RBCs are susceptible to piscine orthoreovirus (PRV) (20), and to infectious salmon anemia virus (ISAV) (21) which can agglutinate RBCs, and replicate within them. The Gram-negative bacterium *Aeromonas hydrophila* can induce ferroptosis or iron-dependent programmed death in catfish RBCs (18). The myxozoan parasite *Sphaerospora molnari* causes hemolytic anemia in common carp and actively feeds on RBCs by incorporating host proteins (19).

Therefore, nucleated RBCs are extremely attractive to study their immunological background, particularly to answer the burning question: whether nucleated RBCs not only enable pathogen replication but also actively resist or defend themselves, such as in a manner demonstrated by Pereiro et al.. The study revealed, that the erythrocytes of the turbot fish permit replication of viral hemorrhagic septicemia virus (VHSV) but simultaneously suppress it in a sophisticated autophagy-dependent manner, potentially via the activity of the antimicrobial peptide Nk-lysin peptide Nk-lysin (22).

In this manuscript, our goal is to further investigate the immunological function of RBCs in a basal fish species: the common carp (*Cyprinus carpio*). In addition, given their abundance, circulation and interaction with every physiological system of the host, we hypothesize that they may act as immune sentinels that are poised to react to immune challenges. To study RBCs from this perspective requires understanding of their response kinetics, their immune repertoire, and any potential direct microbicidal activity *in vitro* and *in vivo*. To accomplish this, we applied various tools, methods, and stimuli that immune effector cells conventionally respond to. We challenged the RBCs with particles, LPS, and *Aeromonas hydrophila*, a ubiquitous fish pathogen, to elicit activities such as cytokine secretion, PRR expression, ROS production, and particle engulfment. Their extensive immune arsenal of effector molecules, PRRs, and microbicidal activities position them as effective immune sentinels that constantly survey the state of the host. Especially when RBCs are directly targeted by pathogens, we must further study evolutionarily distant RBCs alongside WBCs, as they together determine the outcome of any intervention, therapy, prevention strategy, or disease.

## 2 Results

### 2.1 *In vivo* infection with *A. hydrophila* changes the blood cell composition and induces ROS production

**Figure 1:**
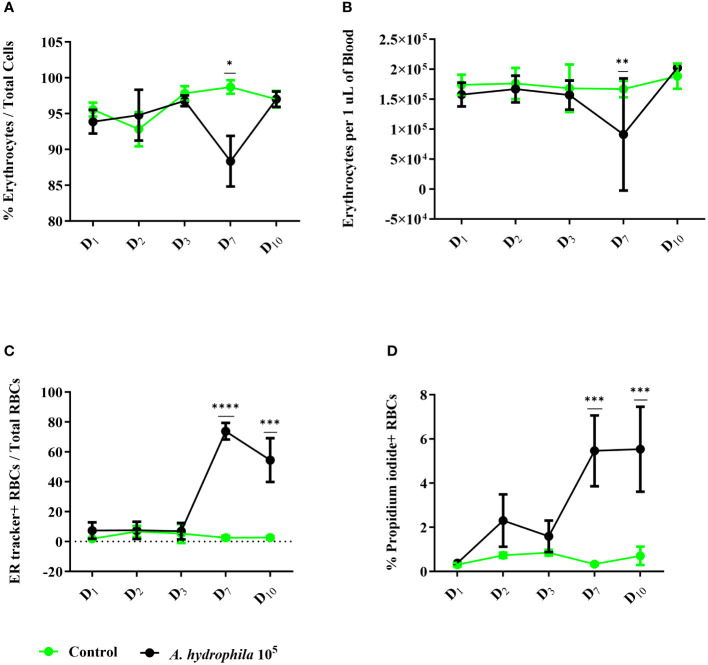
Cellular changes in the red blood cell (RBC) compartment throughout *in vivo* infection with *A. hydrophila*. Healthy carp (n = 14) were divided into two groups. The control group (n = 7) was intraperitoneally injected with 100 μL of RPMI 1640, and the experimental group was challenged with 10^5^ *A. hydrophila* (n = 7). The number of erythrocytes was measured on select days and evaluated by flow cytometry as **(A)** proportion of total cells or **(B)** concentration per μL of blood. We also quantified the proportion and concentration of **(C)** ER tracker+ cells and **(D)** propidium iodide+ dead cells. The data is presented as mean values with SD error bars. A two-way ANOVA was performed with Dunnett’s multiple comparisons *post hoc* test to compare each experimental condition to the respective RPMI group at each time point. * *p*< 0.05; ** *p*< 0.01; *** *p*< 0.001; **** *p*< 0.0001.

To evaluate how RBCs contribute to the immune response, we first performed an infection experiment with *A. hydrophila*. Following an intraperitoneal injection of 100,000 bacterial cells, we evaluated the total number of circulating erythrocytes as well as their phenotype via flow cytometry (**Figure 1**). Throughout the ten days of the experiment, we measured only marginal changes in the RBC compartment which manifested on day 7 by a 10% decline in the proportion of RBCs or a decrease of about 50,000 RBCs per μL in absolute numbers (**Figures 1A, B**). Beginning on day 7, we also measured a steep and sustained increase in the proportion of ER tracker+ RBCs in infected fish, reaching over 70% of all erythrocytes, and a 5% increase in the number of propidium iodide-positive (PI+) dead erythrocytes (**Figures 1C, D**). Our data indicate that although the infection does not induce any dramatic changes in the number of circulating RBCs, it changes the phenotype of these cells towards higher endoplasmic reticulum activity.

### 2.2 *In vivo* bacterial challenge induces expression of pro-inflammatory cytokines

The expansion in translational/endoplasmic reticulum activity indicates that erythrocytes participate in the immune response. Thus, we analyzed changes in the expression of select cytokines that orchestrate inflammatory responses and compared them to the cytokine signatures of WBCs during the initial 10 days of *A. hydrophila* infection.

**Figure 2:**
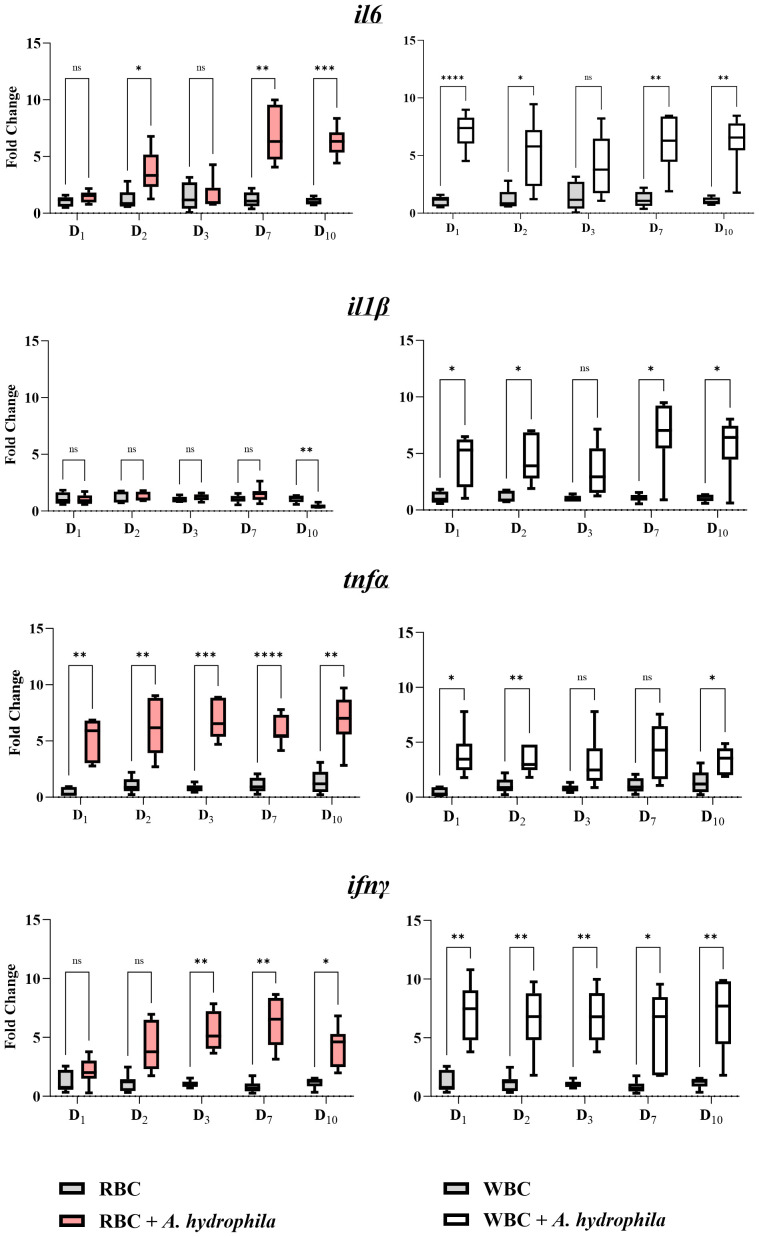
Gene expression profile of red blood cells (RBCs, left column) (n = 7) and white blood cells (WBCs, right column) (n = 7) during an *in vivo* bacterial infection over the course of 10 days. A pair of graphs were created for each target gene (*il6*, *il1β*, *tnfα*, or *ifnγ*). The units of measure are fold changes of the target gene relative to the housekeeping gene (*ef1-α*) (ΔCt) relative to the corresponding unstimulated group (ΔΔCt). Data are depicted as box-and-whisker plots, where the whiskers extend to the smallest and largest value. The line in the middle of the box represents the median. A two-way ANOVA was performed with Dunnett’s multiple comparisons *post hoc* test to compare each experimental condition to the corresponding uninfected group at each time point. ns (not significant); * *p*< 0.05; ** *p*< 0.01; *** *p*< 0.001; **** *p*< 0.0001.

We could not discern any clear trend or pattern in the gene expression of WBCs (**Figure 2**, right column). Except for *ifnγ*, the expression of the three other cytokines did not display significant upregulation on day 3 post-infection, besides *ifnγ*. Among all genes tested, the differential expression of *tnfα* was weakest in the WBCs, although the approximately 3-fold log_2_ changes in expression were statistically significant on days 1, 2 and 10. In comparison, the expression of *il6*, *il1β*, and *ifnγ* were three orders of magnitude higher than *tnfα* at multiple time points.

On the other hand, in the RBCs, gene expression analysis unveiled a significant upregulation of three of the tested cytokines (**Figure 2**, left column) and no increase in *il1β* expression. We observed significant upregulation of *tnfα* at all sampling time points, *ifnγ* and *il6* at three out of five tested time points. Notably, these cytokines were all overexpressed on day 7, corresponding with peak infection in terms of anemia, proportion of dead erythrocytes, and ER expansion (**Figure 1**). Impressively, the expression of *tnfα* and *ifnγ* was over 5 log_2_ units higher relative to the uninfected fish. Furthermore, *il6* expression between days 2 and 10 ranged from about a 3 to 6 log_2_ fold-change compared to the control group. Overall, the responses of RBCs and WBCs exhibited differences in profile, kinetics, and magnitude of expression. Given that all four cytokines have pro-inflammatory properties, our data strongly suggest that common carp RBCs play a role in antibacterial immunity in physiological settings.

### 2.3 *In vitro* stimulation with *A. hydrophila* induces the expression of pro-inflammatory cytokines

**Figure 3:**
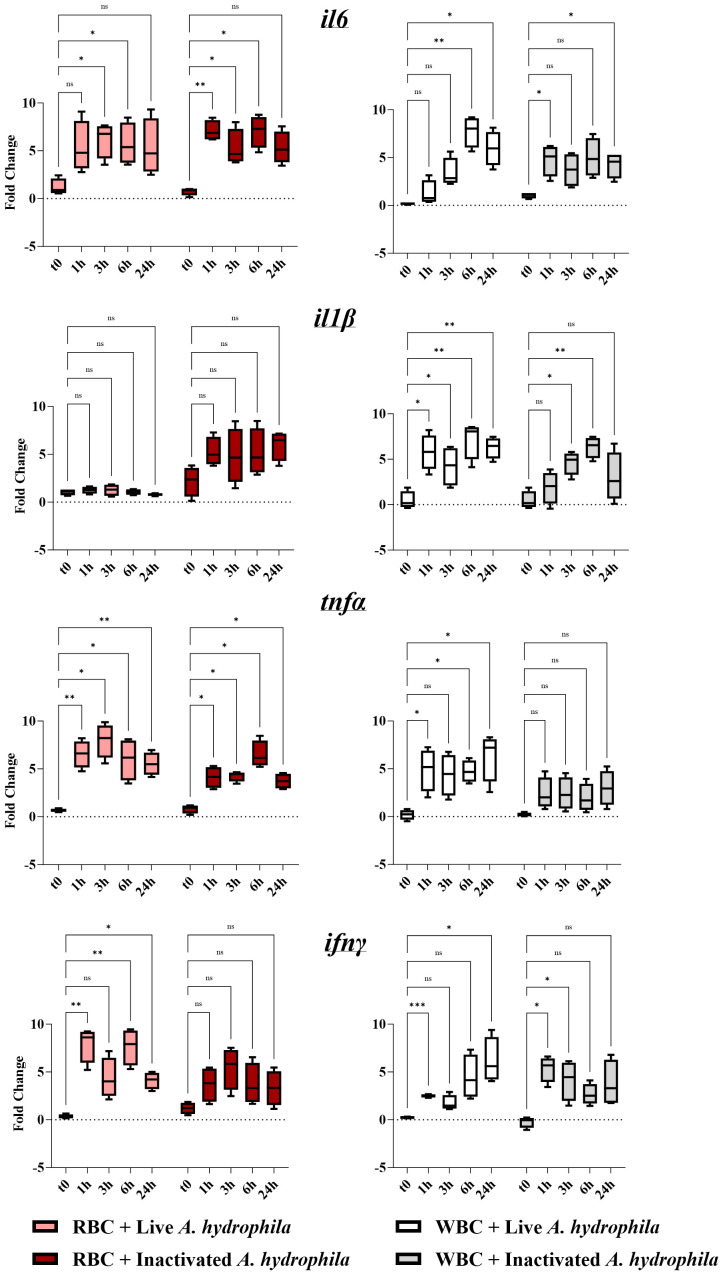
Cytokine gene expression profiles of red blood cells (RBCs, left column) and white blood cells (WBCs, right column) of 7 fish (biological replicates) stimulated with live and inactivated bacteria. A pair of graphs was created for each target gene (*il6*, *il1β*, *tnfα*, or *ifnγ*). The units of measure are fold changes of the target gene relative to the housekeeping gene (*ef1-α*) (ΔCt) relative to the corresponding baseline control (t0) (ΔΔCt). Data are depicted as box-and-whisker plots, where the whiskers extend to the smallest and largest values, and the midline represents the median. A two-way ANOVA was performed with Dunnett’s multiple comparisons *post hoc* test to compare each experimental group to their respective t0 RPMI group at each time point. Annotations indicate statistical significance: ns (not significant); * *p*< 0.05; ** *p*< 0.01; *** *p*< 0.001.

Given the changes in the transcriptional profile of both RBCs and WBCs during *in vivo* infection, we next delineated whether the observed effect in erythrocytes is caused by a paracrine stimulation from activated WBCs and whether RBCs are capable of expressing the cytokines independently. Thus, we performed a series of *in vitro* co-incubation experiments with *A*. *hydrophila* at different time points for both WBCs and RBCs (**Figure 3**). *Ex vivo* RBCs collected prior to incubation (t0) expressed baseline levels of all cytokine genes (**Figure 3**, left column). By exposing the erythrocytes to live *A. hydrophila*, *tnfα* expression was induced at all time points post-stimulation and *ifnγ* was induced at all but the 3h time point, with expression peaking at an over 7-log_2_ change for both genes. The expression of *il6* was significantly different only at the 3h and 6h time point, while *il1β* expression showed no significant difference at any point in time. In contrast, WBCs overexpressed *il1β* at all time points, while *il6* and *ifnγ* were both overexpressed at two time points (**Figure 3**). Unlike the RBCs, *ifnγ* reached a peak in expression 24h post-stimulation. Notably, *tnfα* expression was significantly upregulated in WBCs at all but the 3h time point. Our data indicate that both RBCs and WBCs exhibit peak responses to the bacterium at different time points post-stimulation.

Additionally, to distinguish between a response to the hemolytic/pathogenic activity of live bacteria versus detection of MAMPs on the surfaces of inactivated bacteria, we performed the assay using dead bacteria (**Figure 3**). Upon stimulation with inactivated bacteria, erythrocytes significantly upregulated *il6* as early as 1h post-stimulation (over 6-log_2_ fold-change) and at all but the 24h time point. Although *tnfα* was upregulated at all time points post-challenge, it was weaker than that against live bacteria at its peak. Inactivated bacteria did not significantly upregulate *ifnγ* and *il1β* in these cells. Therefore, in RBCs, *tnfα* and *ifnγ* expression were less sensitive to inactivated bacteria. In WBCs, in comparison to the response to live *A. hydrophila*, the inactivated *A. hydrophila* led to accelerated and weaker *ifnγ* and *il1β* responses respectively. *tnfα* expression was no longer significantly different at any time point post-incubation with live bacteria. Both RBCs and WBCs tended to be less sensitive to inactivated bacteria and could be distinguished by the RBCs’ early and heightened *tnfα, ifnγ* and *il6* expression.

### 2.4 RBCs secrete the cytokines TNFα and IFNγ upon *in vitro* stimulation with *A. hydrophila*

**Figure 4:**
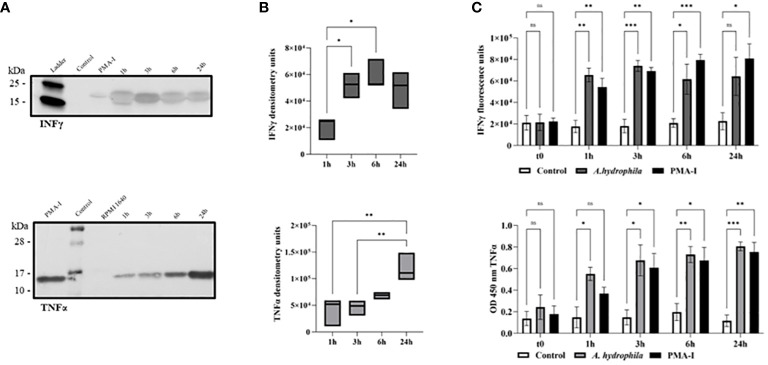
TNFα and IFNγ protein detection by western blotting of monensin-treated RBCs following *in vitro* stimulation with *A*. *hydrophila*. The samples include PMA-ionomycin-stimulated cells (PMA-I) after 24h, mock-stimulated cells (RPMI 1640) after 24h, and *A*. *hydrophila*-stimulated RBCs 1h post-stimulation (1h), 3h post-stimulation (3h), 6h post-stimulation (6h), and 24h post-stimulation (24h). **(A)** TNFα protein was detected at an approximate molecular weight of 16 kDa while IFNγ was detected at 20 kDa in the representative blots of n = 3 independent experiments. **(B)** Densitometry analysis of all biological replicates (n = 3 to 4 independent experiments) is summarized in two box plot graphs. The line in the middle represents median. The data is analyzed using a one-way ANOVA with Tukey’s *post hoc* test. * p< 0.05; ** p< 0.01. **(C)** ELISA detection of secreted TNFα (top) and IFNγ (bottom) in supernatant of cultured RBCs (n = 7 biological replicates for each experimental group) following *in vitro* incubation with *A*. *hydrophila*. The negative control represents the supernatant of the cells incubated with RPMI 1640, while the positive control is the supernatant from cells incubated with PMA-I. A two-way ANOVA was performed with Dunnett’s multiple comparisons *post hoc* test to compare each experimental condition to the respective RPMI 1640 control group at the same corresponding time point. Units are reported in optical density at 450 nm (OD 450 nm) or fluorescence units. Statistical significance is indicated as follows: ns (not significant); * *p*< 0.05; ** *p*< 0.01; *** *p*< 0.001.

Based on gene expression, we expected RBCs to be a potent source of TNFα and IFNγ protein. The western blot revealed that erythrocytes produce the cytokines at all time points upon stimulation with bacteria *in vitro* (**Figures 4A, B**). The absence of any detectable protein 24h after RPMI 1640 mock treatment suggests that the cytokines detected at 1h reflect a rapid response rather than basal levels or pre-formed levels of cytokine. TNFα protein was detected at an approximate molecular weight of 16 kDa, while IFNγ was detected at 20 kDa, corresponding to their theoretical molecular weights. Densitometry (**Figure 4B**) revealed significantly more of both cytokines at later time points (either at 24h for TNFα, or both 3h and 6h for IFNγ) suggesting increased synthesis in response to induced gene expression (**Figure 3**).

After identifying intracellular cytokines within RBCs, we conducted an enzyme-linked immunosorbent assay (ELISA) to confirm protein secretion using the same antibodies described above. Secreted cytokine was detectable as early as 1h post-stimulation (**Figure 4C**). Cytokine levels were significantly different from the negative control at all time points, except for IFNγ at the 24h time point. As ELISA can detect secreted cytokine that has accumulated up to the measured time point, our data suggest that IFNγ secretion ceased by the 3h time point while that of TNFα continued past the 6h time point. In summary, aligning with gene expression data, RBCs detect and respond to Gram-negative bacteria, exhibiting potent cytokine production, particularly of TNFα.

### 2.5 Erythrocytes of common carp produce ROS

**Figure 5:**
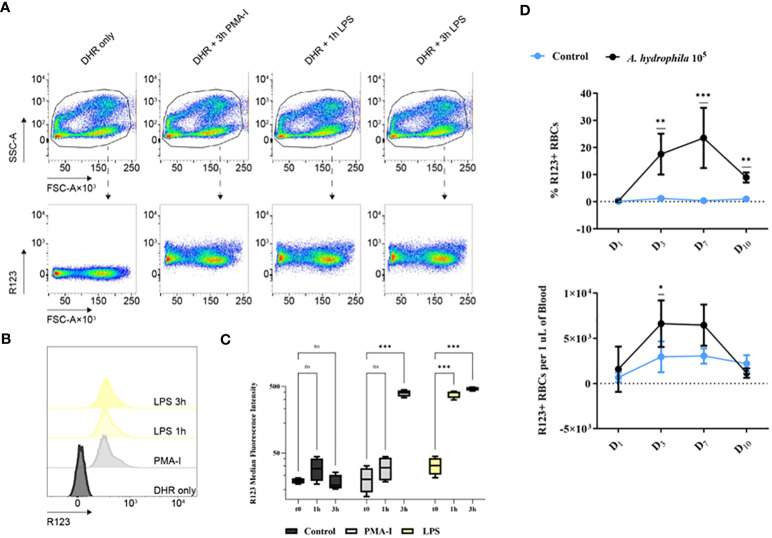
Rhodamine 123-positive (R123-positive) red blood cells (RBCs) analyzed by **(A)** flow cytometry. Plots of R123 fluorescence (y-axis) versus forward scatter area (FSC-A). The samples include: the control group (RPMI 1640-treated and incubated with DHR only), the positive control group (PMA-I), the LPS-stimulated groups after 1h or 3h of stimulation (1h LPS or 3h LPS). **(B)** Representative histograms of the flow cytometry data presented in **(A)** along with the summary of the data **(C)**: MFI for all experimental groups at the tested time points. Data are presented as box-and-whisker plots. The whiskers range from the smallest measured value and up to the largest. The line in the middle of the box is positioned at the median. A two-way ANOVA was performed with Dunnett’s multiple comparisons *post hoc* test to compare each experimental condition to their respective t0 group at each time point. n = 4 biological replicates. **(D)** The proportion and the concentration of R123+ cells from *in vivo A. hydrophila* challenge. The data is presented as mean values with SD error bars. A two-way ANOVA was performed with Dunnett’s multiple comparisons *post hoc* test to compare each experimental condition to the respective RPMI group at each time point. ns (not significant); * *p*< 0.05; ** *p*< 0.01; *** *p*< 0.001.

As live *A. hydrophila* directly interacts with RBCs and consumes hemoglobin, our data with dead bacteria suggests the recognition of a MAMP. To investigate whether RBCs respond to bacterial contact, particularly to lipopolysaccharide (LPS), a universal stimulus and component of Gram-negative bacteria, we conducted flow cytometry analysis. The results revealed significant production of ROS at both time points in the LPS-stimulated group, as indicated by the production of fluorescent rhodamine 123 (R123) (**Figures 5A–C**). We measured the highest R123 fluorescence intensity reflecting the most ROS produced at both time points after LPS stimulation but only after 3h of stimulation with the positive control, stimulation with phorbol myristate acetate-ionomycin (PMA-I) (**Figures 5A–C**). Representative data is shown as density plots and histograms (**Figures 5A, B** respectively) and summarized (**Figure 5C**).

Apart from *in vitro* LPS stimulation we also observed significant ROS production in *in vivo* infection (**Figure 5D**). The proportion of ROS-producing R123+ erythrocytes increased continuously and peaked at 20% on day 7. Our data indicate that the RBCs produce ROS against bacteria not only *in vitro* but also in *in vivo* infection. Despite *A. hydrophila* actively lysing erythrocytes, our findings indicate that RBCs are not defenseless.

### 2.6 Common carp RBCs engulf latex beads

**Figure 6:**
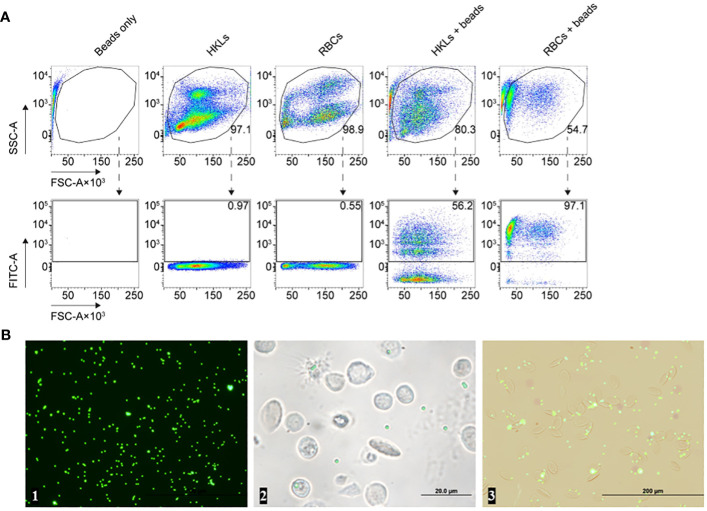
**(A)** Flow cytometry analysis of the head kidney leukocytes (HKLs, positive control), and red blood cells (RBCs), after incubation with RPMI 1640 or carboxylate-modified latex beads. Distinct populations of HKLs, RBCs, and latex beads were identified based on their side scatter area (SSC-A) versus forward scatter area (FSC-A) profiles, as shown in the top row. The bottom row illustrates the level of green fluorescence intensity (y-axes), representing the fluorescence of latex beads in the FITC channel, versus FSC-A (x-axes) for the same corresponding experimental conditions presented in the top row. A single gated subpopulation is included in each plot: host cells in the top row and latex bead-associated host cells in the bottom row. We included the proportion/percentage of each subpopulation out of total events next to their corresponding plots which e.g., enumerates the number of phagocytic HKLs or latex bead-associated RBCs. **(B)** Fluorescence microscopy images of (from left to right): beads only, head kidney leukocytes incubated with beads, and the erythrocytes incubated with beads after 1 hour. These images are derived from the same specimens included in **(A)**.

**Figure 7:**
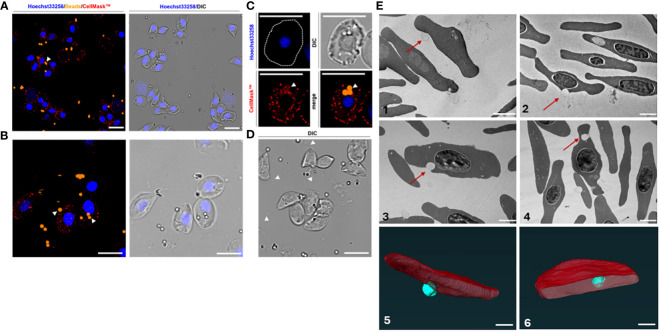
Immunofluorescence of the erythrocytes incubated with carboxylate-modified latex beads **(A, B)**. Erythrocytes were stained with Hoechst33258 (DNA; blue), CellMask (RBC membranes; red), and incubated with latex beads (Alexa Fluor™ 555; orange). All pictures were also captured and merged using differential interference contrast (DIC). Apart from adherence of beads to the surface of RBCs **(A, B)**, beads were also internalized within the membrane accompanied by co-staining with a membrane-specific dye (CellMask™) of the RBCs: **(C)** subcellular presence of beads (indicated by white arrowheads) within an erythrocyte. **(D)** Erythrocytes forming filamentous membrane extensions in the presence of beads (also indicated by white arrowheads). Scale bars: 10 μm. **(E)** Transmission electron microscopy (1-4) and the serial block face-scanning electron microscopy (SBF-SEM) (5-6) of the erythrocytes incubated with carboxylate-modified polystyrene latex beads (0.5 μm) for 1h (1), 2h (2-3) or 4h (4). The electron micrographs show the adhesion of the beads to the erythrocytes (1-2) or engulfment of the beads (red arrows) (3-4). Scale bars: 2 μm. (5-6) Segmented portion of 3D SBF-SEM of bead engulfment by erythrocytes. Organelle segmentation is color-coded. Red: erythrocytes, Turquoise: latex beads. Scale bars: 1µm.

We proceeded to evaluate the capacity of RBCs to attach to and engulf latex beads. Carboxylate-modified fluorescent beads were incubated with RBCs and head kidney leukocytes (HKLs), the latter serving as a positive control, and were subjected to flow cytometry (**Figure 6**), transmission electron microscopy (TEM, **Figure 7E**), confocal microscopy (**Figures 7A–D**), and serial block face-scanning electron microscopy (SBF-SEM) (**Figure 7E**). In the flow cytometry analysis, almost all RBCs were FITC+ while the proportion of FITC+ HKLs was half of that (**Figure 6A**). While WBCs with more than two beads were increasingly rare, on the contrary, RBCs associated with less than two beads or none were a rarity.

Although our results suggest that RBCs had a high propensity for engulfing multiple beads, microscopy rather indicates that many of the beads are surface-level associations and not necessarily internalized latex beads (**Figure 6B**). Confocal microscopy further revealed adhesion of the beads to the membrane of the RBCs (**Figures 7A–C**). Nonetheless, we observed filamentous membrane extensions (**Figure 7D**), and some engulfed particles by TEM and SBF-SEM (**Figure 7E**). Additionally, 3D projections of confocal Z-stacks supported the adherence of beads to the surface of RBCs (**Supplementary Video**).

### 2.7 Common carp RBCs express several putative toll-like receptors for pattern recognition

**Figure 8:**
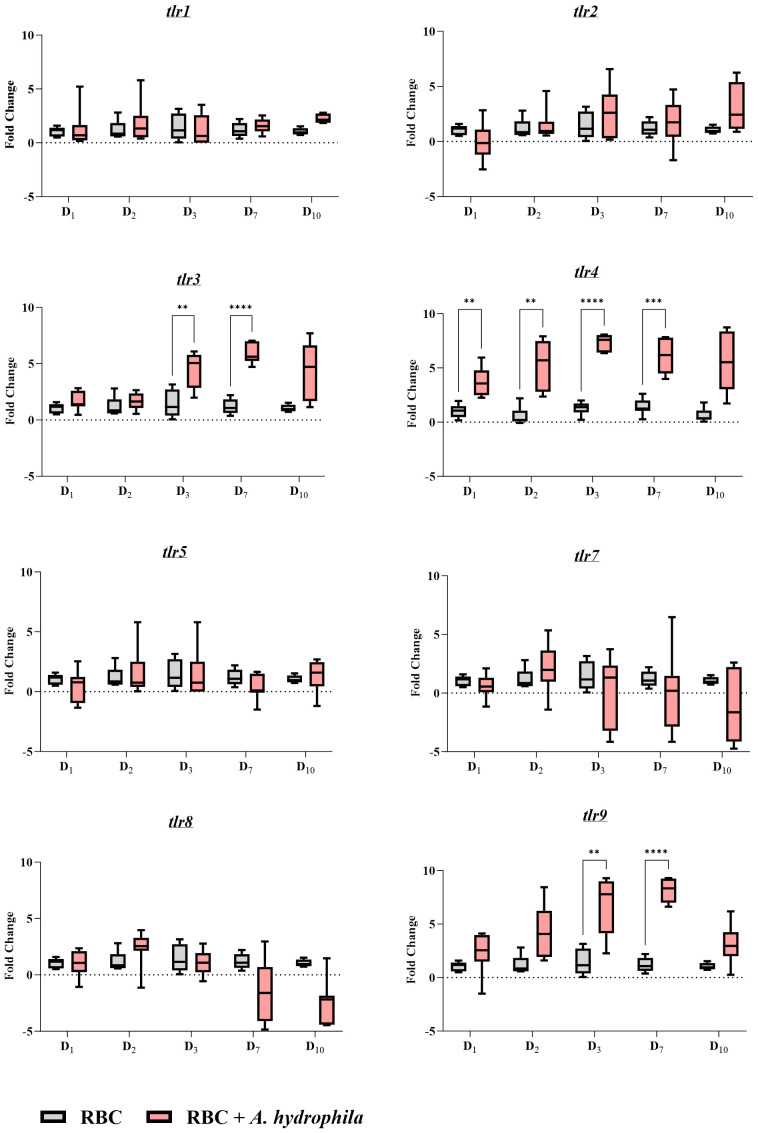
Gene expression profile of toll-like receptors (*tlrs*) during an *in vivo* bacterial infection over the course of a 10-day period. A pair of graphs were created for each target gene as indicated in the graph names. The units of measure are fold changes of the target gene relative to the housekeeping gene (*ef1-α*) (ΔCt) relative to the unstimulated control at the corresponding time point (ΔΔCt). Data are depicted as box plots. The midline of each box represents the median. A two-way ANOVA was performed with Tukey’s *post hoc* test to compare the infected group at a given time point to the corresponding uninfected group at the same time point. ** *p*< 0.01; *** *p<* 0.001; **** *p*< 0.0001. n = 7 biological replicates.

As erythrocytes display strong reactivity to both *in vitro* and *in vivo* bacterial infections, we investigated potential receptors that might underlie these responses. Focusing on TLRs which are PRRs that are activated during infection, we analyzed samples obtained from *in vivo* infections. Our analysis revealed expression of *tlr4* (**Figure 8**), known for binding to LPS. Furthermore, we observed that RBCs also express *tlr9*, a receptor that binds CpG DNA, and *tlr3*, which binds double-stranded RNA (dsRNA) (**Figure 8**).

In the *A. hydrophila* infection group, all three TLRs were significantly upregulated on days 3 and 7, with the latter representing the peak of infection and *tlr* expression. However, only *tlr4* showed significant upregulation on the first two days post-infection, with its expression peaking at an over 7-fold log_2_ change compared to the control group. The expression of *tlr3* and that of *tlr9* was over 5- and 7-fold overexpressed in log_2_ units respectively, on day 7 post-infection. Notably, the expression of the other receptors were unchanged throughout the infection.

## 3 Discussion

### 3.1 Common carp erythrocytes mount a pro-inflammatory response against *A. hydrophila*

Our investigation revealed a pro-inflammatory response against *A. hydrophila* in erythrocytes via expression of the cytokines *tnfα*, *ifnγ*, and *il6*. Against live or inactivated *A. hydrophila*, *in vitro* or *in vivo*, *tnfα* was consistently overexpressed whereas we never measured *il1β* overexpression relative to non-stimulated RBCs. Nucleated rainbow trout RBCs were also reported to synthesize TNFα and IL1β protein (23) and our results indicate that common carp RBCs also produce TNFα. What effect on the immune response can we expect from these cytokines?

It is in the context of leukocytes that cytokine activities are best studied and understood. The cytokine profile we observed from erythrocytes resembles that of zebrafish head kidney leukocytes which upregulate *tnfα, il1β*, and *ifny* after *A. hydrophila* infection (24). Teleost head kidney leukocytes can produce IFNγ in response to LPS alone ​ (25)​. Among all the cytokines, IFNγ stands out in its capacity to induce a T helper 1 response in fish (25)​ which promotes cell-mediated immunity by activating phagocytes to kill intracellular pathogens. Potentially, RBCs may participate in IFNγ-driven polarization of macrophages into the M1 phenotype and subsequently T helper 1 cell differentiation ​ (26)​. Common carp IFNγ induces ROS production/the respiratory burst in macrophages as well as expression of *tnfα* and *il1β* ​ (27)​. The end result is protection against pathogens as exemplified by common carp IFNγ conferring protection against spring viremia of carp virus ​ (28).

Regarding the other cytokines, they are also LPS-inducible in fish (29, 30). In teleosts, TNFα orchestrates antibacterial responses, is prominently produced by macrophages, and induces expression of *ifny* (31)​. Curiously, rainbow trout TNFα broadly inhibited RBC expression of *il6*, *tnfα* itself but also the chemokine *il8*, the PRRs *tlr3* and *tlr9*, as well as genes responsible for antigen presentation (32). As for the IL6, one study of recombinant rainbow trout IL6 observed that it can induce macrophage proliferation as well as production of the antimicrobial peptide hepcidin (30)​​.

### 3.2 Fish RBCs have antimicrobial activity

Beyond acting through or on leukocytes, teleost RBCs may have microbicidal activities of their own. We observed evidence that carp RBCs engulfed particles and produced ROS.

We demonstrated that the RBCs of a teleost fish engulfed μm-sized particles. Interestingly, we were also able to observe the presence of filamentous extensions that are often seen in mammalian macrophages (33). If the carp RBCs indeed phagocytosed the non-opsonized particles, it would be a feature shared with teleost head kidney macrophages as demonstrated by Frøystad et al. (34). Although one study described pseudopodia formed by frog and bird but not fish erythrocytes (35), another observed grass carp RBCs forming pseudopodia albeit *in vitro* (15).

Regardless of whether teleost RBCs are phagocytes, our results pertain only to synthetic latex beads and we need more evidence before we can draw conclusions about bacterial phagocytosis, a process that is microbicidal and activates an immune response. Once taken up, the bacterium must also be broken down. Both mammalian and teleost RBCs oxidize hemoglobin to produce ROS in response to bacterial infection (36, 37). Our data on ROS production *in vivo* and *in vitro* together with published works by Xu et al. (14) and Qin et al. (36)​ suggest that teleost species such as common carp, grass carp, and catfish may be capable of lysosomal activity against bacteria. These two studies also provided evidence of phagocytic activity in grass carp and catfish, as well as demonstrating that grass carp hemoglobin can be activated by LPS and proteolysis.

Finally, it remains unclear if this culminates in processing of exogenous proteins into peptides, antigen presentation, and an immune response. A study of rainbow trout infected with VHSV suggests that their RBCs may express major histocompatibility complex (MHC) class I and II as well as the co-stimulatory molecules CD83 and CD86 (38) whereas another in the rock bream also indicates that teleost RBCs can present antigen (39). However, we lack functional evidence of RBCs deploying these molecules in antigen presentation let alone T cell activation.

### 3.3 RBCs as immune sentinels

From transgenic zebrafish studies, known early responders to infection likely include resident macrophage subsets that are conserved in fish (40, 41), and leukocytes such as neutrophils and macrophages recruited by pathogens (42). Our data suggest that RBCs have the tools to act alongside these other rapid immune responders. RBCs may be uniquely positioned to survey the state of the host given their abundance, circulation, interaction with every physiological system and (from this study) swift response as well as vast immune repertoire.

Interestingly, we demonstrated that the carp erythrocytes secrete TNFα and IFNγ within an hour of *A. hydrophila* stimulation, making them competent early responders. At the protein level, immature mammalian TNFα is cleaved from the plasma membrane (43)​. Curiously, we did not detect TNFα in unstimulated cells by western blotting, suggesting that RBCs synthesize it rapidly *de novo* upon stimulation or that immature TNFα is not recognizable by our antibody.

The expanded immune receptor repertoire of teleost RBCs versus that described so far for mammalian RBCs supports the role of fish RBCs as immune sentinels. In this study, the latex beads may be recognized by scavenger receptors through their carboxylate modification which mimics the surface charge of dead cells in mammals ​ (34). ​This may be useful for trafficking apoptotic cells in homeostasis and in disease for clearance in the spleen or kidney. However, we cannot explain for now the vast difference between RBCs and WBCs in proportion, trend, and profile in this assay (**Figure 6A**). Detection of a bacterium like *A. hydrophila* would be through a different receptor and mechanism. Our data suggests that the common carp expresses an LPS-binding PRR for detection of Gram-negative bacteria. The common carp expresses three paralogues of TLR4, two of which are upregulated by *A. hydrophila* infection and one of which is downregulated (44). However, these receptors and their ligands are poorly characterized. The zebrafish counterpart was demonstrated to bind *A. hydrophila* in one study (45); another demonstrated that TLR4 of several teleost species does not bind LPS (46); a more recent article by Loes et al. (47) observed that one of the TLR4 paralogues of zebrafish signals via ​the Md-2 coreceptor and activates NF-κB, but a separate MyD88-dependent pathway is more important for the response.

In our study, RBCs express at least eight *tlr* genes and three of them are upregulated by *A. hydrophila* including *tlr3* and *tlr9* which may express orthologues of TLRs binding endosomal nucleic acids (48). Gong et al. observed that common carp splenocyte *tlr3*, *tlr4*, and *tlr9* were responsive to *A. hydrophila* (44) whereas Uma et al. measured upregulation of *tlr9* in various organs such as skin, gill, brain, liver, intestine, and kidney of zebrafish (49). With our data, it is now reasonable to assume that RBCs contribute to this expression, at least in the spleen. The regulation of *tlr9* may be via TNFα which can upregulate this *tlr* gene in non-myeloid cells of the gilthead seabream (50). As for evidence of *tlr* expression in fish RBCs: *tlr3* is expressed by poly(I:C)-stimulated tilapia RBCs; other receptors such as *tlr2*, *tlr5* and *tlr9* may also be expressed at low levels in unstimulated RBCs according to transcriptomic data (51); rainbow trout RBCs express both *tlr3* and *tlr9* (32).

Overall, although we demonstrated that common carp RBCs have innate and even isolated immune capacity *in vitro*, it is difficult to gauge their contribution to an immune response relative to that of WBCs because on the one hand, erythrocytes outnumber the WBCs nearly 100 to 1 in fish, and on the other, our expression data does not account for the different reactive and non-reactive subpopulations among the highly heterogeneous leukocytes. Additionally, we must account for erythrocytes responding to their own cytokines or for any (reciprocal) bystander interaction with leukocytes (52) as demonstrated by Jeong et al. in a transwell assay after LPS stimulation which upregulated 338 transcripts in beakfish RBCs relative to an RBC monoculture (39). Furthermore, we do not yet know the context in which RBCs encounter exogenous immunogen but it may be facilitated by cytokine activity: e.g., IL1β activity which induces angiogenesis and is a vasodilator (53)​.

In summary, RBCs are more multi-faceted than was once appreciated. Beyond the immunological activities we directly studied, teleost RBCs can also mount antiviral responses via interferon-stimulated genes and proteins (54). They produce antimicrobial peptides in response to viral infection (13, 22). The nature of the stimulus/pathogen can also elicit cytokine profiles that are the opposite of what we observed (38). Therefore, the immune capacities described so far for teleost RBCs are just the tip of the iceberg. Erythrocytes may be targetable for therapy or prophylaxis as demonstrated by Puente-Marin et al. (32), much like how leukocytes are routinely targeted for host protection. Especially because of their susceptibility to bacterial, viral and parasitic pathogens, all immune cells including RBCs must be studied in unison to understand the overall innate and adaptive immunity of teleost fishes.

## 4 Materials and methods

### 4.1 Experimental animals

We reared specific pathogen-free (SPF) common carp (*C. carpio*) from peroxide-treated fertilized eggs (700 mg/L for 15 min) in an experimental recirculating system in the animal facility of the Institute of Parasitology, Biology Centre CAS. Fish were housed in separate tanks with UV-irradiated and ozonized water at 21 ± 1°C; water quality (oxygen, pH, ammonia, nitrite, and nitrates) was monitored daily using probes and titration tests. Ammonia levels never surpassed 0.02 mg/L. During the experiment, fish with a mass of approximately 25 g were selected and fed twice a day with a commercial carp diet (Skretting) at a daily rate of 1.5% of their body weight.

### 4.2 *A. hydrophila* culture

*A. hydrophila* BSK-10 was obtained from the Department of Evolutionary Immunology, Institute of Zoology and Biomedical Research, Faculty of Biology, Jagiellonian University in Krakow, Poland. These bacteria were isolated from infected carps and selected on an appropriate medium for *Aeromonas* (Rimler-Shotts Agar) by the Polish Academy of Sciences, Institute of Ichthyobiology and Aquaculture in Golysz in Poland. This strain has already been used in previous studies and has been shown to cause immunological changes (55–57).

Bacteria were grown in the laboratory in Luria-Bertani (LB) agar and LB broth at 37°C for 24h. Next, bacteria were centrifuged at 1600 *g* for 10 min, and the bacterial pellet was reconstituted in sterile PBS (280 mOsM). Optical density was measured at 625 nm, and data were aligned with a previously derived McFarland scale to determine the bacterial concentration as done previously by Maciuszek et al. and Falco et al. (58, 59).

Additionally, PCR was performed with selected *Aeromonas hydrophila-specific* primers of *16S rRNA* for additional confirmation of the bacterial species and the purity of the strain used for the performed experiments (**Supplementary Figure 1**).

For the purpose of the stimulation experiment with inactivated *A.hydrophila*, the bacterium was inactivated with Intracellular (IC) Fixation Buffer (Thermo Fisher Scientific, USA) for 15 min at room temperature. The bacterial pellet of 1 x 10^5^ CFU/mL was resuspended in 150 µL of the fixation buffer. The bacterium was afterwards washed three times in 1x PBS buffer by centrifugation for 5 min at 500 *g*.

### 4.3 Blood separation, RBC/WBC sampling

We collected 500 µL of whole blood from 7 individual fish using syringes rinsed with heparin at a concentration of 5000 IU per mL. The fish were previously anesthetized in clove oil. The blood was diluted in the cell culture medium RPMI 1640 (Gibco, USA) at a ratio of 1:4. We then layered the blood on top of 1.077 g/mL Ficoll-Paque PREMIUM medium (Cytiva, Sweden) for density centrifugation and separation of RBCs from WBCs. Centrifugation was for 10 min at 500 *g* with minimal acceleration and deceleration/braking (i.e., both adjusted to ‘1’ on the centrifuge). This short program increased purity of the RBC/WBC fractions, while reducing the time spent between blood collection and experimenting with the cells. We collected either the pellet containing the erythrocyte fraction or the buffy layer enriched for leukocytes. To ensure the purity of both WBC and RBC suspensions, we repeated the density centrifugation. The samples underwent additional confirmation of purity through both flow cytometry and light microscopy by blood smear analysis (**Supplementary Figure 2**). Subsequently, these freshly processed individual cell suspensions were used for downstream assays.

### 4.4 *A. hydrophila in vitro* stimulation assay

Freshly isolated and separated RBCs and WBCs were obtained from 7 SPF carps. The cells were first counted in Bürker chambers under an Olympus light microscope, and the cell concentrations were adjusted to 1 x 10^6^ RBCs or WBCs per mL of RPMI 1640 before being added to a non-tissue culture-treated 24-well plate. Isolated RBCs and WBCs were added individually and separately to their corresponding wells. *A. hydrophila*, maintained in LB broth, was pelleted, re-suspended in RPMI 1640, and quantified. 1 x 10^5^ CFU/mL *A. hydrophila* were added to all suspensions of cells, except for the negative control. The experimental conditions included: i) RBCs/WBCs simply cultured in RPMI 1640, ii) RBCs/WBCs challenged with live bacteria and iii) RBCs/WBCs challenged with formaldehyde-inactivated bacteria. Where applicable, cells were also stimulated with Cell Stimulation Cocktail (PMA-I) (Thermo Fisher Scientific, USA), serving as a positive control (60). Both RBCs and WBCs were plated individually and separately. The cells of each biological replicate were plated in their own corresponding wells without mixing. The cells were incubated for 1h, 6h, or 24h at 26.5°C, CO_2_ 5%. Afterwards, the cells were harvested, centrifuged at 500 *g* for 5 min at 4 °C and the pellets were collected for immediate RNA isolation.

### 4.5 *A. hydrophila in vivo* infection

To evaluate the RBC and WBC immune response *in vivo*, prior to the experiment, 14 fish (weight = ± 25 g) were individually tagged with glass transponders (AEG). At the start of the experiment, fish were divided into two groups: control (CO) (n = 7) fish were intraperitoneally injected with 100 µL of sterile RPMI 1640 and the other group was infected (n = 7) (INF). Infection was achieved by intraperitoneal injection with 1 x 10^5^ A*. hydrophila* CFU/mL resuspended in 100 µL of RPMI 1640. All fish were anesthetized with 0.1 mL/L of clove oil in water before bleeding or injection. Sampling of fish was performed 1, 2, 3, 7 and 10 days post-infection. On each sampling day, 200 µL of blood was drawn with a heparinized syringe and diluted in the cell culture medium RPMI 1640 (Gibco, USA) at a ratio of 1:4. We then separated the cells by density as described in Materials and Methods 4.4. Additionally, 7 naïve fish were sampled on day 0 (before any injections) to establish a baseline and to measure the immune activity of the RBCs in the absence of any stimulation. The samples underwent additional confirmation of purity through both flow cytometry and light microscopy (**Supplementary Figure 2**). Subsequently, these freshly processed individual cell suspensions were directly used for RNA isolation and cDNA synthesis.

### 4.6 Evaluation of RBC ER activity, viability, ROS production and phagocytic activity by flow cytometry

To evaluate the purity of the isolated cells and to track changes in the number and the activity of RBCs throughout the *in vivo* infection, the sampling of the blood was always followed by flow cytometry analysis. The number of erythrocytes and the composition of the blood was determined. Briefly, 2 µL of whole blood was resuspended in 200 µL of RPMI 1640. Each sample was acquired for 20 seconds on a FACSCanto II (BD Biosciences, USA) at a flow rate of 60 µL/min. Erythrocytes were identified based on the forward scatter width (FSC-W) and side scatter area (SSC-A). The ER also plays a significant role during bacterial infection, where the data indicate that bacteria have evolved strategies to differentially activate arms of ER stress sensors resulting in specific host cell responses (61). Additionally, increased ER activity has been detected upon LPS stimulation in the teleost fish (62). In order to study ER activity, we used ER-tracker Green (BODIPY FL Glibenclamide) (Thermo Fisher Scientific, USA): a cell-permeant stain that binds to the sulphonylurea receptors of ATP-sensitive K+ channels prominent in the ER. 1 µL of ER-tracker was added to 999 µL of 1x HBSS buffer. The solution was added to the cells for 15 min at 27°C. To estimate the amount of erythrocyte death, cells were labelled with propidium iodide (Sigma Aldrich, Germany). A total of 10 µL of propidium iodide was added to each 1 mL of cell suspension. The cells were incubated for 10 min at room temperature. Finally, to detect the production of ROS, cells were incubated for 15 min at 27°C with DHR123 (dihydrorhodamine 123; Thermo Fisher Scientific, USA) at a concentration of 10 mM, whereby ROS oxidizes DHR123 into fluorescent R123.

For the purpose of *in vitro* ROS detection, 1 × 10^6^ RBCs/mL were stimulated with LPS at a concentration of 50 µg/mL: the cells were resuspended in 1 mL of RPMI 1640 and incubated for 1 or 3 hours. The cells were also stimulated with PMA-I, serving as a positive control. For the detection of ROS, cells were incubated for 15 min at 27°C with DHR123.

For the phagocytic activity assay, 5 × 10^6^ RBCs/mL and 1× 10^6^ HKLs/mL in individual tubes were incubated with carboxylate-modified polystyrene fluorescent yellow-green latex beads, 0.5 μm or 1 μm (Sigma Aldrich, USA) or pHrodo green *E. coli* BioParticles (Thermo Fisher Scientific, Germany) at a ratio of 1:10 (cells to beads). Following the incubation for 1h at 28°C, the cells were washed four times with 1x PBS and the phagocytosis of RBCs was evaluated.

### 4.7 Western blotting

Erythrocyte lysates of 1 × 10^6^ cells were collected throughout *in vitro* stimulation: 1, 3, 6, and 24 hours post-bacterial inoculation. Each experimental group of cells was treated with monensin (3 µM) for 4h at 27°C, 5% CO_2_ (Thermo Fisher Scientific, USA), an inhibitor of intracellular protein transport. The cells were prepared in Laemmli sample buffer (Bio-Rad Laboratories, USA), in Mini-PROTEAN TGX precast gels 4-20% (Bio-Rad, USA), and transferred to Immuno-Blot PVDF membranes (Bio-Rad, USA), pre-activated in methanol. Two membranes were blocked for 1 hour at room temperature in 7% bovine serum albumin (BSA) (Thermo Fisher Scientific, USA) in Tris-buffered saline, 0.1% Tween 20 (TBST). After blocking, for TNFα detection, we incubated membranes with polyclonal anti-zebrafish TNFα antibody (Kingfisher Biotech, Inc, USA) at 1:1000 while others used for the detection of IFNγ were incubated with the anti-IFNγ-Alexa Fluor 594 (N3-P3A5*A10) against zebrafish (Novus Biologicals, USA) at 1:1000, added to 5% BSA in TBST, overnight at 4°C. The membranes were then washed three times for 7 min per wash with TBST, and incubated with secondary antibodies in 5% BSA. To detect TNFα, we used goat anti-rabbit IgG (H + L chains) conjugated to horseradish peroxidase (Invitrogen, Germany) at 1:5000 incubated for 1 hour at room temperature. We detected any bound primary anti-IFNγ antibody using goat anti-mouse IgG conjugated to Alexa Fluor 647 (Thermo Fisher Scientific, USA) at 1:5000 in TBST and incubated for 1 hour at room temperature. The membranes were washed three times for 5 min per wash and exposed to Clarity Western ECL Substrate solution (Bio-Rad, USA) to detect TNFα. The chemiluminescent signal was documented using ChemiDoc MP Gel Imaging System (Bio-Rad, USA) with the optimal ‘auto exposure’ setting.

### 4.8 ELISA

To evaluate whether gene expression translates to secretion of TNFα and IFNγ, we collected the supernatant from RBCs after 1, 3, 6, and 24 hours of incubation with bacteria. The supernatant was additionally diluted in sodium carbonate-bicarbonate buffer and coated onto two 96-well flat-bottom EIA/RIA plates (Corning Incorporated, Costar, USA) overnight at 4°C. The next day, the plates were washed 3 times with PBS 0.1% Tween 20 (PBST) and left in blocking buffer for 3 hours. The blocking buffer was washed away before the anti-zebrafish TNFα polyclonal antibody (Kingfisher Biotech, Inc, USA) and anti-IFNγ-Alexa Fluor™ 594 antibody (N3-P3A5*A10) (Novus Biologicals, USA) were added to two separate plates (one for each cytokine) at 1:2000 and 1:1000, respectively. For detection of TNFα, the plate was washed 3 times before addition of the goat anti-rabbit IgG (H + L chains) secondary antibody conjugated to HRP (Invitrogen, Germany) at 1:5000. After a 1-hour incubation at room temperature, the secondary antibody was washed away and TMB substrate (Bio-Rad, USA) was added for the detection of the signal. The reaction was stopped by the addition of 2M sulfuric acid. The OD was measured on a TECAN plate reader (Life Sciences, USA) at 450 nm wavelength. Fluorescence was measured from the second plate (used for IFNγ detection) with the Spark® Multimode Microplate Reader (Life Sciences, USA) at emission wavelength 594 nm.

### 4.9 Phagocytic activity

Following the previously mentioned protocol, the phagocytosis of RBCs was evaluated in four additional approaches, besides flow cytometry. For this purpose we have used fluorescence microscopy, TEM, confocal microscopy, and SBF-SEM.

For TEM, 5 × 10^6^ cells were fixed with glutaraldehyde and centrifuged at 1800 *g*. Fixed cells were then frozen with a Leica EM PACT2 high pressure freezer (Leica Microsystems). Using a Leica AFS (Leica Microsystems), samples were freeze-substituted in 100% acetone containing 2% OsO4 for 96 hours at −90 °C. Temperature was raised 5 °C/h to −20 °C and after 24 hours, samples were rinsed in acetone and infiltrated in graded series of resin (EMBed 812, EMS) solutions (25%, 50% 75% in acetone) 1 hour each. Cells were infiltrated in pure resin overnight, embedded in fresh resin and polymerized at 60 °C for 48 hours. Ultrathin sections were stained with uranyl acetate and lead citrate and examined either by JEOL 200 kV 2100 F or JEOL JEM-1010 microscopes. Dual-axis tilt series was collected in the range of ±65° with 0.6°-increments using a 200 kV JEOL 2100 F TEM equipped with a high-tilt stage and Gatan camera (Orius SC 1000) and controlled by SerialEM automated acquisition software.

For fluorescence confocal microscopy, the cell suspension with the latex beads was stained with Hoechst (Invitrogen) for 15 minutes at 27°C. After incubation the cells were washed three times with 1x PBS and the CellMask Deep Red (ThermoScientific, Czech Republic) was added as a membrane stain for 30 minutes on ice. The cells were washed with 1x PBS and spun down with a cytospin centrifuge directly with the microscopic slides (ThermoScientific, Czech Republic). The slides were observed with an Olympus FluoView™ FV1000 confocal microscope. Negative controls omitting the primary antibodies were carried out and were consistently negative.

For the SBF-SEM, the sample preparation by the high-pressure freezing technique followed the protocol for TEM sample preparation. After freeze-substitution, the samples were subsequently stained with 1% thiocarbohydrazide in 100% acetone for 1.5 hours, 2% OsO_4_ in 100% acetone for 2 hours at room temperature, and 1% uranyl acetate in 100% acetone overnight at 4°C. After every staining step, the samples were washed 3 times with 100% acetone for 15 min. Samples were then infiltrated with 25%, 50%, or 75% acetone-resin mixture for 2 hours at each step, and finally infiltrated in 100% Hard Resin Plus 812 (EMS) overnight and polymerized at 62°C for 48 hours. Resin-embedded blocks were trimmed and imaged using an Apreo SEM equipped with a VolumeScope (Thermo Fisher Scientific, Germany). Serial images were acquired at 3.5 keV, 50 pA, 40 Pa with a resolution of 6 nm, 100 nm slice thickness, and dwell time per pixel of 4 μs. The analysis of the images and the 3D model was done on Microscopy Image Browser (MIB MATLAB 2.84) (63)​ software and Amira (Thermo Fisher Scientific, USA) platform for visualization, processing, and analysis of 3D models.

### 4.10 qPCR and gene expression analysis

To evaluate inflammatory responses after *in vivo* and *in vitro* stimulation, we measured expression of cytokines *tnfα*, *il1β*, *il6a*, *ifnγ* in the erythrocytes and the leukocytes by real-time PCR. Moreover, the same set of RBC samples obtained from *in vivo* infection were used to detect *tlrs* expressed by the erythrocytes in the bacterial infection. For this purpose, we measured the expression of the *tlr1*, *tlr2*, *tlr3*, *tlr4*, *tlr5*, *tlr7*, *tlr8* and *tlr9* genes. Expression of these target genes were calculated relative to the housekeeping gene Elongation factor 1-alpha (*ef1-α)*. Primer sequences are presented in **Supplementary Table 1**. We prepared RNA freshly from RBCs and WBCs using the RNeasy Mini Kit (Qiagen, Hilden, Germany) according to the manufacturer’s instructions. The integrity of RNA was evaluated using the Agilent 2100 Bioanalyzer (Agilent Technologies, USA) and the Eukaryote Total RNA Nano Assay (Agilent Technologies, USA) revealing RIN values ranging from 20 to 100. A total of 10 ng of RNA per specimen was subjected to Transcriptor High Fidelity cDNA Synthesis (Roche, Germany) according to the manufacturer’s recommendations, and using the recommended thermocycler program. NTC (non-template control) and a non-RT (-RT, non-reverse transcriptase) were included. Technical duplicate measurements were made on the QuantStudio 6 (Applied Biosystems, USA). PCRs were performed with 2 μL of 10–20-fold diluted cDNA, 10 μL of Fast SYBR Green Master Mix and 0.4 μM of each specific primer set in 20 μL mixtures. Discrepancies of over a half cycle between technical duplicates were addressed by repeating the same specimens in a new reaction/plate, adjusting Ct values based on an inter-run calibrator, and retaining only replicates with low standard deviations. The data was analyzed using Pfaffl method (64) using the formula:


$$Ratio=\;\frac{{\left(E_{target}\right)}^{\Delta Ct}Target^{\left(control-sample\right)}}{{\left(E_{reference}\right)}^{\Delta Ct}Reference^{\left(control-sample\right)}}$$

where E is the efficiency of the primers for each gene, target represents the gene of interest and reference is the housekeeping gene *ef1-α.*

### 4.11 Statistical analysis

The data were analyzed using the software Prism 10 (GraphPad Software, USA). In the gene expression graphs, the data are presented as mean values ± standard deviation (SD). Statistical analyses were conducted on fold change gene expression data calculated by the Pfaffl method. Gene expression data for *in vitro* and *in vivo* experiment were analyzed using two-way ANOVA with Dunnett’s multiple comparisons *post hoc* test to compare each experimental condition to the corresponding uninfected group at each time point. Gene expression data for *tlrs* was analyzed by two-way ANOVA with Tukey’s *post hoc* test to compare the infected group at a given time point to the corresponding uninfected group at the same time point. Western blot densitometry analysis of all biological replicates is analyzed using a one-way ANOVA with Tukey’s *post hoc* test. The statistical test applied for each assay is indicated in their respective figure legends.

## Data availability statement

The original contributions presented in the study are included in the article/**Supplementary Material**. Further inquiries can be directed to the corresponding author.

## Ethics statement

The animal study was approved by the Resort Professional Commission of the CAS for Approval of Projects of Experiments on Animals. The manipulation and sampling protocols were executed with a consistent approach and in strict adherence to the provisions of the Czech legislation governing the welfare of animals, as set forth in the Protection of Animals Against Cruelty Act No. 246/1992. All procedures were authorized by the Czech Ministry of Agriculture. The study is reported in accordance with ARRIVE guidelines (https://arriveguidelines.org). The study was conducted in accordance with the local legislation and institutional requirements.

## Author contributions

JM: Conceptualization, Data curation, Formal analysis, Investigation, Methodology, Visualization, Writing – original draft, Writing – review & editing. JK: Data curation, Formal analysis, Methodology, Writing – original draft, Writing – review & editing. KK: Formal analysis, Methodology, Software, Writing – review & editing. MM: Data curation, Formal analysis, Software, Supervision, Writing – review & editing. JC: Conceptualization, Data curation, Formal analysis, Investigation, Methodology, Supervision, Visualization, Writing – original draft, Writing – review & editing. TK: Conceptualization, Data curation, Formal analysis, Methodology, Supervision, Writing – review & editing. AH: Conceptualization, Data curation, Formal analysis, Funding acquisition, Investigation, Methodology, Project administration, Resources, Software, Supervision, Validation, Visualization, Writing – review & editing.
