# Supplementary material for: Erythrocytes of the common carp are immune sentinels that sense pathogen molecular patterns, engulf particles and secrete pro-inflammatory cytokines against bacterial infection
Source: Front Immunol. 2024 Jun 14;15:1407237. doi: 10.3389/fimmu.2024.1407237 (PMC11211254; doi:10.3389/fimmu.2024.1407237)
Supplement: Supplementary file 1 [file DataSheet_1.docx]

Supplementary Material

# Supplementary Figures and Tables


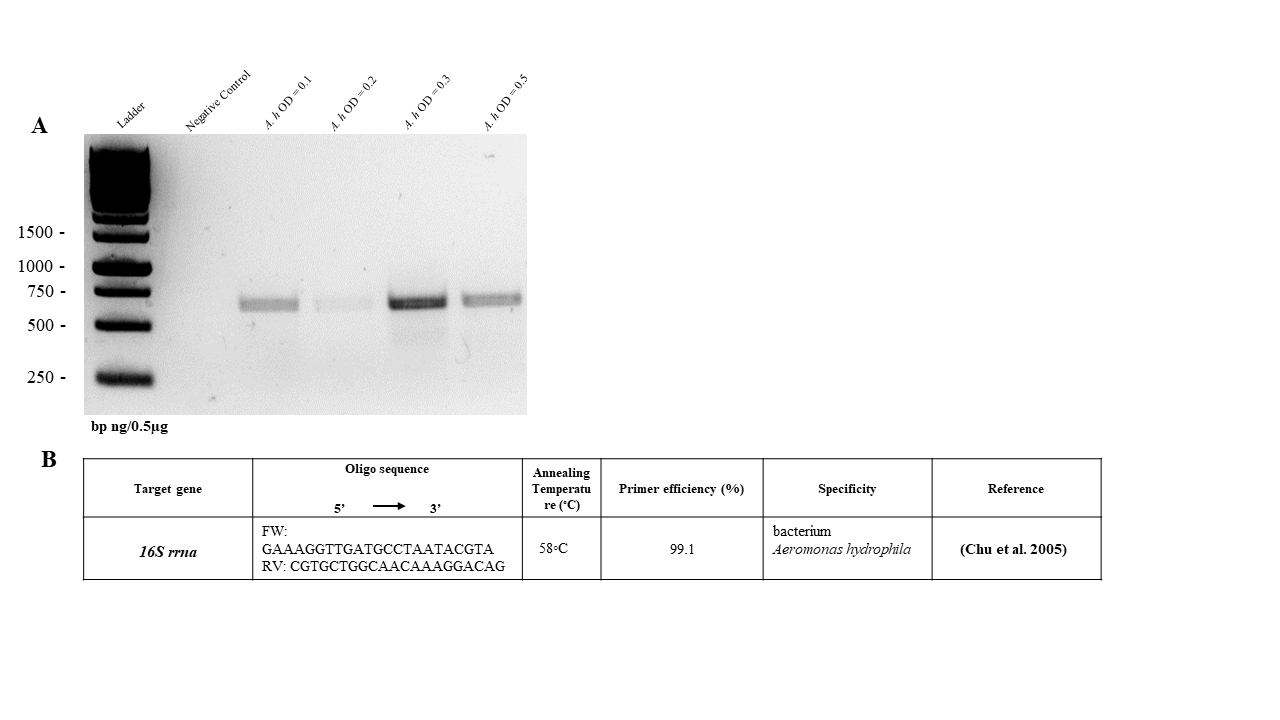
Figure 1. (A) The 16S rRNA PCR using *A. hydrophila* primers. lane (1): peqGOLD 1kb DNA ladder protein , lane (2): negative control without template DNA; lanes 3, 4, 5 and 6: PCR products of *Aeromonas* isolates with different optical density. (B) Specifications of primer sequences used for *A. hydrophila* identification

Figure 2. Blood smear of post-Ficoll separation medium centrifugation of the red blood cells (RBCs) (1-5) and the white blood cells (WBCs) of five biological replicates. Each biological replicate has an image of the RBCs (up) with corresponding WBCs (down). The samples were stained using Hemacolor (Sigma Aldrich, Germany) protocol and the images were obtained on the a fluorescence microscope (Olympus BX51 light microscope).  
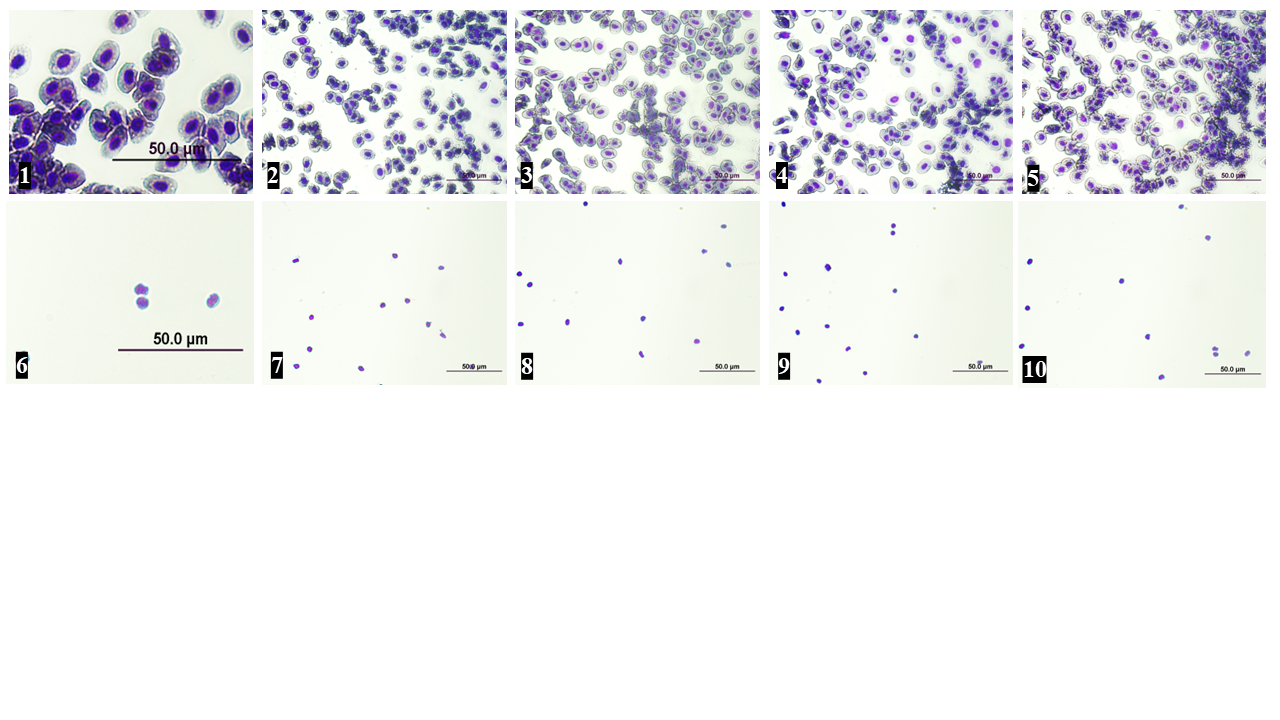


Table 1. RT-qPCR primers sequences

| **Target gene** | **Oligo sequence**  **5’ 3’** | **Annealing Temperature (°C)** | **Primer efficiency (%)** | **Specificity** | **Reference** |
| --- | --- | --- | --- | --- | --- |
| ***ef1-α*** | FW: GACAACCCCAAGGCTCTCAA  RV: ACAGCAAAGCGACCAAGAGG | FW: 57.6  RV: 58.4 | 97.3 | fish | **(Yamaguchi et al., 2013)** |
| ***il6*** | FW: CAGATAGCGGACGGAGGGGC  RV: GCGGGTCTCTTCGTGTCTT | FW: 63.7  RV: 57.4 | 94.3 | fish | **(Baloch et al., 2023)** |
| ***il-1β*** | FW: AAGGAGGCCAGTGGCTCTGT  RV: CCTGAAGAAGAGGAGGCTGTCA | FW: 61.6  RV: 58.6 | 97.5 | fish | **(Baloch et al., 2023)** |
| ***tnfα*** | FW: GCTGTCTGCTTCACGCTCAA  RV: CCTTGGAAGTGACATTTGCTTTT | FW: 58.2  RV: 53.3 | 94.9 | fish | **(Ribeiro et al., 2010)** |
| ***ifnγ*** | FW: CGATCAAGGAAGATGACCCAGTC  RV: GTTGCTTCTCTGTAGACACGCTTC | FW: 57.3  RV: 57.8 | 98.4 | fish | **(Embregts et al., 2019)** |
| ***tlr1*** | FW: ATCTACAGCAGACGGAAAG  RV: TCTTGAAGCCCCTGTGAAAG | FW: 57.2  RV: 56.7 | 97.9 | fish | **(Fink et al., 2016)** |
| ***tlr2*** | FW: GAACCTTGTAGGAAACCCAT  RV: CCCATCTAAGCCATTCTTGT | FW: 57.2  RV: 56.7 | 98.7 | fish | **(Wei et al., 2016)** |
| ***tlr3*** | FW: CTGTCTTCCTTGCTTTTGTACTCG  RV: CCCAGTTTAGCAGATTTCAGTTTGT | FW: 56.7  RV:57.4 | 98.4 | fish | **(Wei et al., 2016)** |
| ***tlr4*** | FW: GGCTATAGATCACCTGGACAGC  RV: CCAGACTTCATCATAGCTGGAGA | FW:57  RV: 57.2 | 97.6 | fish | **(Balog et al., 2022)** |
| ***tlr5*** | FW: TTGACGGCTCTGTTGTCCTC  RV: CACGTAAGTCACATGCGTAGG | FW: 56.8  RV: 56.5 | 99.1 | fish | **(Balog et al., 2022)** |
| ***tlr7*** | FW: GGGAATGCAATGAGCCAGA  RV: GAAGAGAACATCAAATCCAGACGA | FW:57.4  RV: 57.5 | 96.2 | fish | **(Wei et al., 2016)** |
| ***tlr8*** | FW: GACTCAACCCTGCGATATACATGA  RV: GCCATTCATGGAGAGATTGAGGCA | FW: 56.4  RV: 57.4 | 97.4 | fish | **(Shan et al., 2018)** |
| ***tlr9*** | FW: CCGGAATGTTGTGTCCCTCA  RV: GCAAGGCTGTGGTTTGACAG | FW:57.4  RV:57.1 | 98.2 | fish | **(Kongchum et al., 2011)** |

## References

Baloch, A. A., Steinhagen, D., Gela, D., Kocour, M., Piačková, V., & Adamek, M. (2023). Immune responses in carp strains with different susceptibility to carp edema virus disease. *PeerJ*, *11*, e15614. https://doi.org/10.7717/peerj.15614

Balog, K., Bagi, Z., Tóth, B., Hegedűs, B., Fehér, M., Stündl, L., & Kusza, S. (2022). Association study between relative expression levels of eight genes and growth rate in Hungarian common carp (Cyprinus carpio). *Saudi Journal of Biological Sciences*, *29*(1), 630–639. https://doi.org/10.1016/j.sjbs.2021.09.036

Chu WH, Lu CP. Multiplex PCR assay for the detection of pathogenic *Aeromonas hydrophila*. Journal of Fish Diseases. 2005 Jul;28(7):437-41.

Embregts, C. W. E., Tadmor-Levi, R., Veselý, T., Pokorová, D., David, L., Wiegertjes, G. F., & Forlenza, M. (2019). Intra-muscular and oral vaccination using a Koi Herpesvirus ORF25 DNA vaccine does not confer protection in common carp (Cyprinus carpio L.). *Fish & Shellfish Immunology*, *85*, 90–98. https://doi.org/10.1016/j.fsi.2018.03.037

Fink, I. R., Pietretti, D., Voogdt, C. G. P., Westphal, A. H., Savelkoul, H. F. J., Forlenza, M., & Wiegertjes, G. F. (2016). Molecular and functional characterization of Toll-like receptor (Tlr)1 and Tlr2 in common carp ( Cyprinus carpio ). *Fish & Shellfish Immunology*, *56*, 70–83. https://doi.org/10.1016/j.fsi.2016.06.049

Kongchum, P., Hallerman, E. M., Hulata, G., David, L., & Palti, Y. (2011). Molecular cloning, characterization and expression analysis of TLR9, MyD88 and TRAF6 genes in common carp (Cyprinus carpio). *Fish & Shellfish Immunology*, *30*(1), 361–371. https://doi.org/10.1016/j.fsi.2010.11.012

Ribeiro, C. M. S., Pontes, M. J. S. L., Bird, S., Chadzinska, M., Scheer, M., Verburg-van Kemenade, B. M. L., Savelkoul, H. F. J., & Wiegertjes, G. F. (2010). Trypanosomiasis-Induced Th17-Like Immune Responses in Carp. *PLoS ONE*, *5*(9), e13012. https://doi.org/10.1371/journal.pone.0013012

Shan S, Liu R, Jiang L, Zhu Y, Li H, Xing W, Yang G. Carp Toll-like receptor 8 (Tlr8): an intracellular Tlr that recruits TIRAP as adaptor and activates AP-1 pathway in immune response. Fish & shellfish immunology. 2018 Nov 1;82:41-9.

Wei, X., Li, X. Z., Zheng, X., Jia, P., Wang, J., Yang, X., Yu, L., Shi, X., Tong, G., & Liu, H. (2016). Toll-like receptors and interferon associated immune factors responses to spring viraemia of carp virus infection in common carp (Cyprinus carpio). *Fish & Shellfish Immunology*, *55*, 568–576. https://doi.org/10.1016/j.fsi.2016.05.043

Yamaguchi T, Katakura F, Someya K, Dijkstra JM, Moritomo T, Nakanishi T. Clonal growth of carp (Cyprinus carpio) T cells in vitro: long-term proliferation of Th2-like cells. Fish & shellfish immunology. 2013 Feb 1;34(2):433-42.

|  |  |  |  |  |  |
| --- | --- | --- | --- | --- | --- |
|  |  |  |  |  |  |
|  |  |  |  |  |  |
|  |  |  |  |  |  |
|  |  |  |  |  |  |
|  |  |  |  |  |  |
|  |  |  |  |  |  |

## pplementary Figures

**Supplementary Figure 1.** The figure legends are required to have the same font as the main text, 12 point normal Times New Roman, single spaced. Please use a single paragraph for each legend and prepare the figures keeping in mind the PDF layout.
